# Inhibition of Post-Synaptic Kv7/KCNQ/M Channels Facilitates Long-Term Potentiation in the Hippocampus

**DOI:** 10.1371/journal.pone.0030402

**Published:** 2012-02-13

**Authors:** Milos M. Petrovic, Jakub Nowacki, Valeria Olivo, Krasimira Tsaneva-Atanasova, Andrew D. Randall, Jack R. Mellor

**Affiliations:** 1 Medical Research Council Centre for Synaptic Plasticity, School of Physiology and Pharmacology, University of Bristol, Bristol, United Kingdom; 2 Bristol Centre for Applied Nonlinear Mathematics, Department of Engineering Mathematics, University of Bristol, Bristol, United Kingdom; 3 Institute of Medical Physiology, School of Medicine, Belgrade University, Beograd, Serbia; Sackler Medical School- Tel Aviv University, Israel

## Abstract

Activation of muscarinic acetylcholine receptors (mAChR) facilitates the induction of synaptic plasticity and enhances cognitive function. In the hippocampus, M_1_ mAChR on CA1 pyramidal cells inhibit both small conductance Ca^2+^-activated KCa2 potassium channels and voltage-activated Kv7 potassium channels. Inhibition of KCa2 channels facilitates long-term potentiation (LTP) by enhancing Ca^2+^calcium influx through postsynaptic NMDA receptors (NMDAR). Inhibition of Kv7 channels is also reported to facilitate LTP but the mechanism of action is unclear. Here, we show that inhibition of Kv7 channels with XE-991 facilitated LTP induced by theta burst pairing at Schaffer collateral commissural synapses in rat hippocampal slices. Similarly, negating Kv7 channel conductance using dynamic clamp methodologies also facilitated LTP. Negation of Kv7 channels by XE-991 or dynamic clamp did not enhance synaptic NMDAR activation in response to theta burst synaptic stimulation. Instead, Kv7 channel inhibition increased the amplitude and duration of the after-depolarisation following a burst of action potentials. Furthermore, the effects of XE-991 were reversed by re-introducing a Kv7-like conductance with dynamic clamp. These data reveal that Kv7 channel inhibition promotes NMDAR opening during LTP induction by enhancing depolarisation during and after bursts of postsynaptic action potentials. Thus, during the induction of LTP M_1_ mAChRs enhance NMDAR opening by two distinct mechanisms namely inhibition of KCa2 and Kv7 channels.

## Introduction

Activation of hippocampal mAChRs by synaptically-released acetylcholine promotes the induction of LTP at glutamatergic synapses. Elimination of this cholinergic activity by lesions or pharmacological interventions results in cognitive deficits. Furthermore, loss of cholinergic function is implicated in disease processes, for example, the progressive cognitive decline in Alzheimer's disease.

The M_1_ subtype of mAChR is a prime candidate to mediate these cholinergic effects due to its ubiquitous expression in the cortex and hippocampus. Learning, working memory and the induction of synaptic plasticity are all impaired in M_1_ receptor knockout mice [1], [2], [3]. Furthermore, M_1_ mAChR specific agonists facilitate LTP induction [4], [5], [6] and improve cognitive function in animal models [7].

The facilitation of LTP by mAChR activation is thought to be mediated by enhancement of synaptic NMDAR opening either by direct alteration of NMDAR channels [8], [9], [10], [11] and/or indirectly by modulation of cellular excitability. mAChRs inhibit a variety of potassium channels including small conductance calcium-activated KCa2 channels (also known as SK channels) [6], [12] and voltage-activated Kv7 channels (also known as KCNQ or M channels) [13], [14], [15]. Activation of M_1_ receptors leads to inhibition of these two channels by molecularly distinct pathways. KCa2 channels are inhibited by activation of protein kinase pathways [6], [12] whereas Kv7 channels are inhibited by a local depletion of PIP2 [16], [17], [18], [19].

KCa2 channels form feedback loops with NMDARs in dendritic spines and ultimately shape excitatory post-synaptic potentials (EPSPs) and the induction of LTP [20], [21], [22], [23], [24], [25]. Therefore their regulation by M_1_ receptors can at least partially explain the facilitation of LTP by acetylcholine [6].

Kv7 channels are voltage-dependent and partially open at the resting membrane potential. Consequently Kv7 inhibition increases both cellular input resistance and the afterdepolarising potential (ADP) that follows single or bursts of action potentials [26], [27], [28]. Pharmacological inhibition of Kv7 channels also facilitates the induction of LTP [29], [30], [31]. An increase in input resistance reduces the attenuation of back-propagating action potentials into the dendrites [32] which together with an increase in ADP enhances postsynaptic depolarisation during and after postsynaptic action potentials. It is proposed that this will facilitate NMDAR activation and therefore LTP induction during coincident presynaptic and postsynaptic firing.

Here we find that inhibition of Kv7 channels facilitates LTP at the Schaffer collateral synapse by enhancing depolarisation during and after postsynaptic action potentials. Thus, M_1_ receptor activation facilitates LTP by dual distinct mechanisms namely inhibition of both KCa2 and Kv7 channels.

## Materials and Methods

### Slice Preparation

All experiments in this study were performed in accordance with UK Home Office guidelines and were approved by the Home Office Licensing Team at the University of Bristol (ref UB/09/011).

Brain slices were prepared from P13–15 male Wistar rats. Following a lethal dose of anaesthetic (isoflurane inhalation), brains were removed and dissected in ice-cold aCSF (in mM, 119 NaCl, 2.5 KCl, 1 NaH_2_PO_4_.H_2_O, 26.2 NaHCO_3_, 10 glucose, 2.5 CaCl_2_, 1.3 MgSO_4_) saturated with 95% O_2_ and 5% CO_2_. Parasaggital hippocampal slices 300–400 µm thick were cut using a vibratome (VT1200, Leica, Germany) and slices were incubated in aCSF at 36°C for 30 minutes following which they were stored at room temperature until use. Before being transferred to the recording chamber the connections between CA3 and CA1 were cut.

### Whole-cell patch clamp recording

Slices were placed in a submerged recording chamber perfused with aCSF (as above) at room temperature with the addition of 50 µM picrotoxin. CA1 pyramidal cells were visualised using infra-red DIC optics on an Olympus BX-50 microscope. Patch clamp pipettes of resistance 4–5 MΩ were pulled from borosillicate filamented glass capillaries (Harvard Apparatus) using a vertical puller (PC-10, Narashige, Japan). Pipettes were filled with an intracellular solution consisting of (in mM) 120 KMeSO_3_, 10 HEPES, 0.2 EGTA, 4 Mg-ATP, 0.3 Na-GTP, 8 NaCl, 10 KCl and set to pH 7.4, 280–285 mOsm.

Recordings from CA1 pyramidal neurons were made with a Multiclamp 700A amplifier (Molecular Devices, USA), filtered at 4 kHz and digitised at 10 kHz using a data acquisition board and Signal acquisition software (CED, Cambridge, UK). Cells were voltage clamped at −70 mV. Series resistance was monitored throughout the experiments and cells that showed >20% change were discarded from subsequent analysis. Recordings were also rejected from analysis if the series resistance was greater than 30 MΩ. Bridge mode recording was employed for all current clamp experiments. Membrane potentials were not corrected for liquid junction potential (calculated as −13.1 mV).

Synaptic responses were evoked in control and test pathways with 100 µs square voltage steps applied at 0.1 Hz through two bipolar stimulating electrodes (FHC) located in *stratum radiatum*. The test pathway was proximal to, and the control pathway distal to, the pyramidal cell layer. Average baseline EPSC amplitudes in control and test pathways were similar for all LTP experiments. Postsynaptic action potentials were initiated through somatic current injections (2 nA, 2 ms) that reliably induced action potentials in all conditions.

The resting membrane potential and input resistance of the cell were monitored in current clamp for a stable baseline period of 10–20 minutes before XE-991 was washed into the recording chamber. The membrane potential and input resistance were monitored for a further 10–20 minutes. Input resistances were measured in response to an 80 pA hyperpolarising current after the membrane voltage reached steady state, and thus included a component resulting from activation of I_h_.

CGP55845, D-AP5, baclofen and picrotoxin were purchased from Tocris. XE-991 was purchased from Ascent Scientific.

### Induction of synaptic plasticity

EPSCs were recorded in voltage clamp from two independent pathways. A theta burst stimulation (TBP) protocol was applied after the neurons were switched into current clamp mode within 10 minutes of entering the whole-cell configuration to prevent wash-out of plasticity. The TBP protocol consisted of three trains of 10 bursts where each burst consisted of five stimuli at 100 Hz. The interburst frequency was 5 Hz. The three trains were separated by 10 second intervals. Where plasticity experiments were carried out in the presence of XE-991, the drug was washed into the bath before the whole cell configuration was achieved and perfused throughout the experiment.

### Dynamic clamp

Dynamic clamp methods were used to both subtract a Kv7 current-like conductance and to reintroduce a Kv7 like conductance following XE-991 application (a rescue protocol). For these experiments we used a Cambridge Conductance scalable DSP-based system SM-2 [33]. The injected M-current conductance was modelled using Hodgkin-Huxley formalism [34] as a non-inactivating current described by the equation
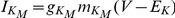
where 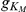 is a maximal conductance of the current, 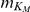 is the activation of the current, i.e. the fraction of channels being open, V is membrane potential of the cell and 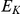 is the reverse potential of the current, which we set to 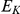 = −85 mV. The gating variable is described by the ordinary differential equation as follows
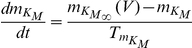
The steady-state activation function 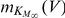 is described using a single Boltzmann function, namely
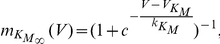
where 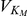 = −30 mV is half activation of the current and 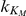 = 10 mV is the slope of the function. The current activation time constant is 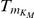 = 75 ms. The values of the current parameters were derived from experimental data taking into consideration junction potential differences between our experiments and published data from other labs [35], [36], [37]. The value of the maximal conductance 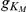, which corresponds to the number of channels present in the cell, often varies. Therefore, in rescue protocols this value was adjusted in every experiment to match the membrane resting potential of the cell to that observed before the M-channel blocker XE-991 was applied.

### Data analysis

Sweeps from the test and control pathways were separated and 6 consecutive traces were averaged together to produce a mean response every minute. EPSC amplitude measurements were taken from the mean traces and normalised to the mean baseline EPSC amplitude. Data are plotted as the mean ± SEM.

Statistical tests were performed using paired or unpaired Student's t-tests as appropriate. LTP was assessed by comparing the mean normalised EPSC amplitudes in control and test pathways 25–30 minutes after induction.

## Results

### Inhibition of Kv7 channels facilitates LTP

**Figure 1:**
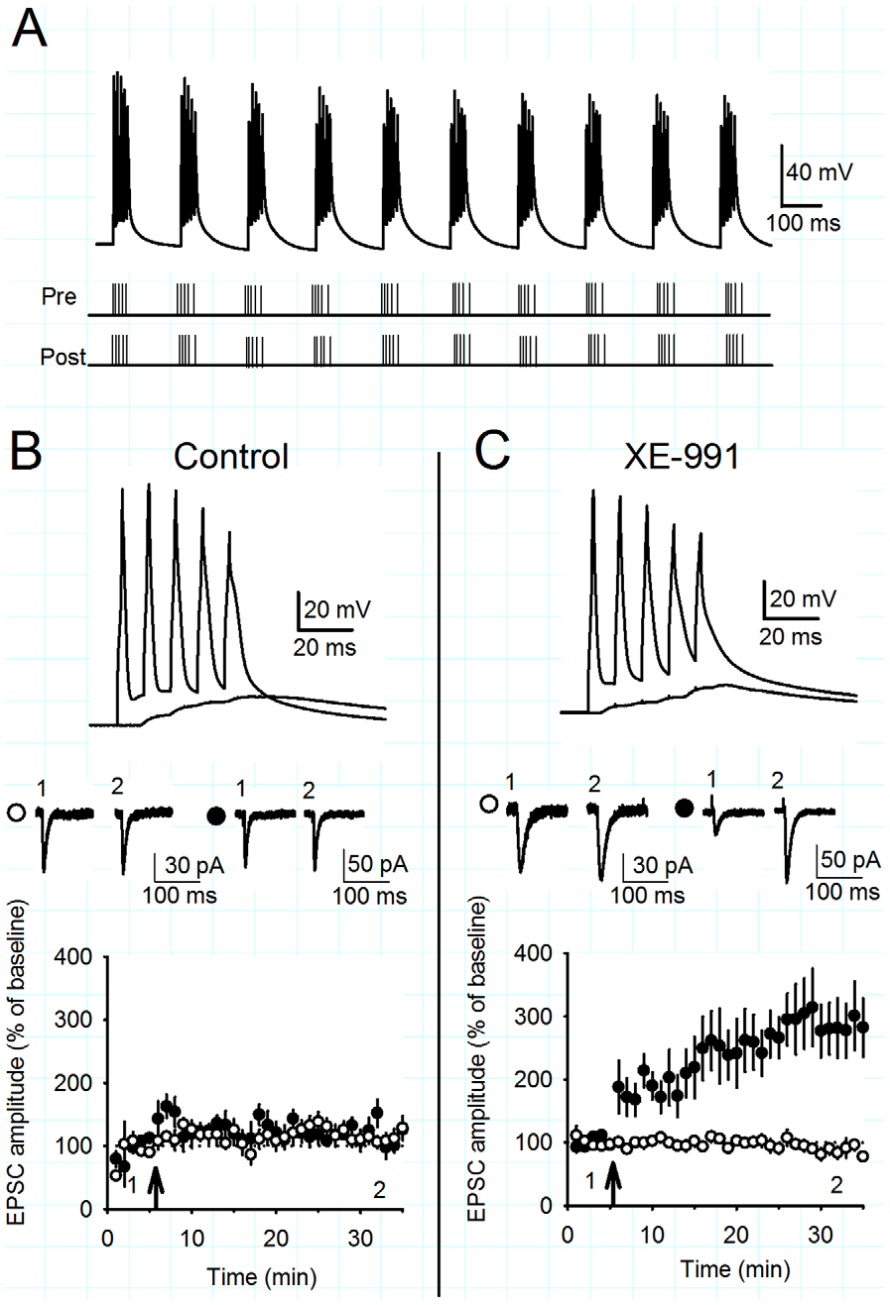
XE-991 facilitates the induction of LTP by theta burst pairing. A) Diagram of theta burst pairing (TBP) protocol. Top, voltage trace of TBP protocol recorded at the soma of a CA1 pyramidal cell. Middle and bottom traces illustrate the timing of inputs to the stimulating and recording electrodes evoking EPSPs and somatic action potentials respectively. B) TBP does not induce LTP under control conditions. Coincident TBP of subthreshold EPSPs and somatic action potentials induced no change in EPSC amplitude in the test (black circles) or control (white circles) pathways. The arrow indicates the timing of the TBP protocol. Example voltage traces show the initial burst of 5 coincident EPSPs and action potentials and a single test burst of 5 subthreshold EPSPs. Example current traces from a single experiment illustrating the mean EPSC response during the baseline (1) and at 30–35 minutes (2) in the test and control pathways. C) TBP does induce LTP in the presence of the Kv7 channel inhibitor XE-991. In the presence of XE-991 (10 µM), coincident TBP of subthreshold EPSPs and somatic action potentials induced pathway-specific LTP.

To investigate the role of Kv7 channels in the induction of LTP at Schaffer collateral commissural synapses we first made use of the Kv7 channel inhibitor XE-991. We have previously shown that a theta burst pairing (TBP) protocol does not induce LTP when excitatory postsynaptic potential (EPSP) amplitude is kept below threshold for the initiation of post-synaptic action potentials but can induce LTP when EPSPs are either suprathreshold [38] or if the NMDAR-mediated component of EPSPs is enhanced by inhibition of KCa2 channels [6]. These findings indicate that TBP with subthreshold EPSP amplitudes is just below the threshold for LTP induction. During LTP experiments, excitatory postsynaptic current (EPSC) amplitude was recorded in voltage clamp in two independent Schaffer collateral pathways. In current clamp, TBP was then applied by pairing stimulation to one of the input pathways with initiation of back-propagating action potentials (b-APs) in the postsynaptic cell. Recordings were then returned to voltage clamp mode to measure the EPSC amplitude in the two input pathways. Importantly, two input pathways were used in all plasticity experiments to rule out non-specific changes in synaptic strength. In addition, in all experiments baseline EPSC amplitudes were small (mean 29.5±9.8 pA, n = 6) to avoid suprathreshold summation of EPSPs during TBP (mean peak summated EPSP 9.4±3.2 mV, mean resting potential −66.9±0.1 mV, n = 6) (Figure 1A; see methods for full description of induction protocol). In agreement with our previous observations no LTP was induced by TBP (Figure 1B; 114±11% vs. 120±10%, control vs. test pathway, n = 6, p>0.05). In contrast, in the presence of 10 µM XE-991, robust test-pathway specific LTP was induced (Figure 1C; 88±5% vs. 284±45%, control vs. test pathway, n = 6, p<0.01) indicating that induction of LTP is facilitated by inhibition of Kv7 channels.

### Inhibition of Kv7 channels does not enhance NMDA EPSPs

**Figure 2:**
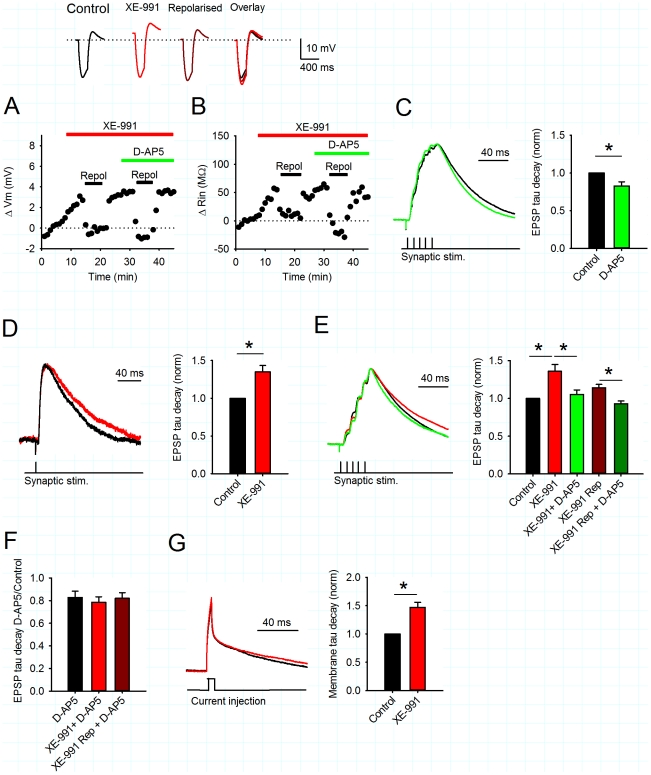
XE-991 does not enhance the NMDAR-mediated component of EPSPs. A) XE-991 (10 µM) depolarised the membrane potential and B) increased input resistance. Example voltage traces show response to a hyperpolarising current in control (black), XE-991 (red), after repolarisation and traces overlaid. Graphs illustrate the timecourse of a single experiment. C) Summated EPSPs during synaptic theta burst stimulation have a small NMDAR-mediated component. Example voltage traces show a burst of five EPSPs under control conditions (black) and in the presence of 50 µM D-AP5 (green). The average normalised decay time constant for a burst of five EPSPs is reduced in the presence of D-AP5. D) XE-991 prolonged the duration of single EPSPs. Example voltage traces in control (black) and XE-991 (red). Average decay time constants increase after application of XE-991. E) XE-991 prolonged the duration of summated EPSPs which was partially reversed either by repolarisation or application of D-AP5. Example voltage traces in control (black), XE-991 (red), XE-991 repolarised (dark red), XE-991 and D-AP5 (green) or XE-991 and D-AP5 repolarised (dark green). Average decay time constants show a partial reversal of EPSP prolongation by D-AP5 at both depolarised and repolarised potentials. F) The effect of D-AP5 on the decay time constant of summated EPSPs was similar in control conditions and in the presence of XE-991. G) XE-991 prolonged the membrane decay time constant in response to a short subthreshold current injection. Example voltage traces in control (black) or XE-991 (red). Average membrane decay time constant shows an increase in XE-991.

Kv7 channels are voltage-dependent potassium channels that contribute to the resting membrane potential of CA1 pyramidal cells and are found primarily on perisomatic membranes [39], [40]. Inhibition of Kv7 channels typically depolarises hippocampal pyramidal neurons and increases their input resistance [26], [27], [41]. In current clamp mode, bath application of 10 µM XE-991 caused a depolarisation of 2.2±0.4 mV (Figure 2A; n = 6, p<0.05) and an increase in input resistance of 53.8±9.6 MΩ (Figure 2B; n = 6, p<0.05). The mean initial resting membrane potential and input resistance were −72.2±1.3 mV and 282.3±31.4 MΩ, respectively (n = 6).

It is reported that Kv7 channels can also regulate presynaptic release probability [42], [43]. We tested this by recording AMPA receptor-mediated EPSCs in voltage clamp with a paired pulse protocol (interstimulus interval 50 ms). Application of 10 µM XE-991 did not change the EPSC amplitude or the paired pulse ratio (PPR) (EPSC amplitude 82.2±10.9%, PPR 98.6±5.4%, n = 5, p>0.05). Subsequent application of the GABA_B_ receptor agonist baclofen (10 µM) that depresses the probability of glutamate release decreased the EPSC amplitude and increased PPR (EPSC amplitude 30.5±11.8%, PPR 205.9±67.4%, n = 5, p<0.05). Thus, we could not detect any effect of Kv7 channels on presynaptic function at the Schaffer collateral synapse in CA1.

The depolarisation and increase in input resistance caused by the inhibition of Kv7 channels could enhance NMDAR activation during synaptic transmission and in particular during TBP, thereby leading to a facilitation of LTP. To assess the component of the EPSP mediated by NMDARs during TBP, 5 presynaptic stimuli were given at 100 Hz and the resulting summated EPSP waveform compared in the presence and absence of 50 µM D-AP5. These experiments were performed in the presence of the GABA_A_ receptor antagonist picrotoxin (50 µM) and the GABA_B_ receptor antagonist CGP55845 (1 µM). EPSP amplitude was set to ensure that EPSP summation was of a similar magnitude to that used in the experiments shown in Figure 1. Under these control conditions, D-AP5 decreased summated EPSP decay time indicating a small NMDAR-mediated component of the EPSP (Figure 2C; decay time constant normalised to control, 0.83±0.06, n = 6, p<0.05). Application of XE-991 caused a prolongation of single EPSPs (Figure 2D; decay time constant normalised to control, 1.35±0.09 in XE-991) and summated EPSPs which was only partially reversed by application of D-AP5 (Figure 2E; decay time constant normalised to control, 1.36±0.09 in XE-991 and 1.05±0.06 with addition of D-AP5, n = 6, p<0.05). When the membrane potential was repolarised to the membrane potential prior to XE-991 addition, D-AP5 still reduced the summated EPSP decay constant, indicating that the XE-991-mediated EPSP prolongation and partial reversal by D-AP5 were not due to membrane depolarisation (Figure 2E; decay constant normalised to control 1.14±0.04 in XE-991 and 0.93±0.04 with addition of D-AP5, n = 6, p<0.05). Comparison of the change in summated EPSP decay time constant with addition of D-AP5 in control conditions or after addition of XE-991 revealed a similar effect of D-AP5 in all conditions (Figure 2F; relative change of decay time constant in D-AP5 0.83±0.06 for control, 0.78±0.05 for XE-991 and 0.82±0.05 for XE-991 repolarised, n = 6, p>0.05) indicating that inhibition of Kv7 channels does not enhance the gating of synaptic NMDARs during EPSPs.

The increase in input resistance caused by inhibition of Kv7 channels would also be expected to increase the membrane time constant of CA1 pyramidal cells providing an alternative mechanism for the prolongation of EPSPs by XE-991. The membrane time constant of the cell, measured by short subthreshold current injections, was also increased by application of XE-991 (Figure 2G; 47.2±3.2 ms in control, 69.1±6.0 ms in XE-991, n = 6, p<0.05). Therefore, we conclude that inhibition of Kv7 channels prolongs EPSPs by increasing the membrane time constant and not by enhancing synaptic NMDAR activity.

### Manipulation of Kv7 conductance by dynamic clamp mimics or reverses the effects of XE-991

**Figure 3:**
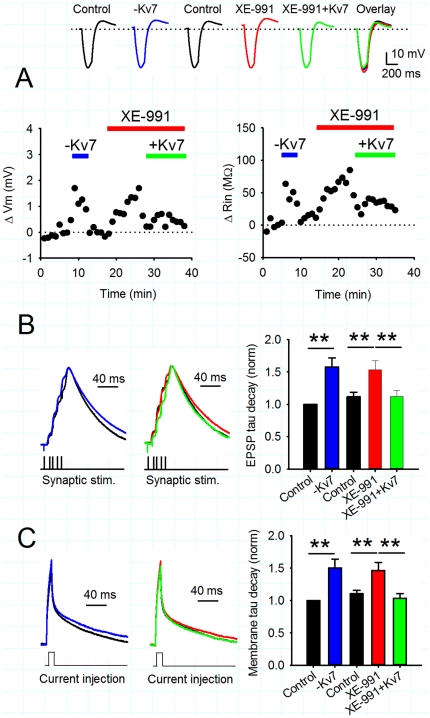
Injection or removal of Kv7 conductance by dynamic clamp reverses or mimics the effects of XE-991. A) Changes in membrane potential and input resistance caused by introduction of negative dynamic clamp Kv7 conductance (blue bar), XE-991 (10 µM, red bar) and positive dynamic clamp Kv7 conductance (green bar). Example voltage traces show responses to a hyperpolarising current in each condition. B) Left: Negative dynamic clamp Kv7 conductance prolongs the duration of summated EPSPs. Switching the dynamic clamping off completely reversed its effects. Example voltage traces in control (black) or negative dynamic clamp (blue). Middle: XE-991 prolonged the duration of summated EPSPs and this effect was completely reversed by positive dynamic clamp Kv7 conductance. Example voltage traces in control (black), XE-991 (red) and XE-991+Kv7 conductance (green). Right: Average membrane decay time constant shows similar increase in both negative dynamic clamp Kv7 conductance and XE-991. The effect of XE-991 is completely reversed by positive dynamic clamp Kv7 conductance. C) Left: Negative dynamic clamp Kv7 conductance prolonged the membrane decay time constant in response to a short subthreshold current injection. Switching the dynamic clamping off completely reversed its effects. Example voltage traces in control (black) or negative dynamic clamp Kv7 conductance (blue). Middle: XE-991 (red) prolonged the membrane decay time constant compared to control (black) and this effect was completely reversed by positive dynamic clamp Kv7 conductance (green). Right: Average membrane decay time constant shows similar increase in both negative dynamic clamp Kv7 conductance and XE-991. The effect of XE-991 is completely reversed by positive dynamic clamp Kv7 conductance.

We next sought to confirm the role of Kv7 like channels in changes to synaptic responses using a dynamic clamp system [33], [44], [45], [46]. This apparatus was used to negate Kv7 conductance and mimic the effect of pharmacological inhibition of Kv7 channels. A model for Kv7 channel voltage dependence and kinetics calculated from experimentally determined values for channels composed of Kv7.2 and Kv7.3 (thought to be the dominant subtypes in CA1 pyramidal cells) was used to parameterize the dynamic clamp system [36], [37], [47], [48] (see methods). To negate existing Kv7 channel activity, the dynamic clamp was used to introduce negative Kv7 conductance to the cell. The amount of conductance was set to generate a depolarisation of ∼2 mV, similar to that produced by pharmacological block of Kv7 channels with XE-991. This also produced an increase in input resistance of 53.6±10.5 MΩ, similar to that produced by XE-991. In each experiment we then switched off the dynamic clamp and applied XE-991 to check that the negation of Kv7 conductance by dynamic clamp and inhibition of Kv7 channels by XE-991 had equivalent effects on membrane potential and input resistance for each cell. Finally, we reversed the effects of XE-991 by using the dynamic clamp system to apply a positive Kv7 conductance with the. This produced a return to baseline values for both membrane potential and input resistance (Figure 3A; ΔVm = 0.06±0.15 mV and ΔRin = 20.3±9.3 MΩ, relative to control values prior to XE-991 application).

The negation of Kv7 conductance with dynamic clamp produced a fully reversible prolongation of summated EPSPs (Figure 3B; decay constant normalised to control 1.58±0.14 fold, n = 7, p<0.01) and an increase in the membrane time constant (Figure 3C, normalised to control 1.50±0.13 fold, n = 7, p<0.01). These increases were similar to those produced by XE-991 (Figure 3B & C; EPSP decay constant normalised to control 1.53±0.14 fold, n = 7, p<0.01 and membrane time constant normalised to control 1.46±0.13 fold, n = 7, p<0.01). When positive Kv7 conductance was applied to replace the conductance inhibited by XE-991, a complete reversal of the EPSP prolongation and membrane time constant increase was observed (Figure 3B & C; summated EPSP decay constant normalised to control 1.12±0.10 fold, n = 7, p<0.01 compared to XE-991 and membrane time constant normalised to control 1.03±0.07 fold, n = 7, p<0.01 compared to XE-991). These data show that input or removal of Kv7 conductance from CA1 pyramidal cells can reverse or mimic the effects of the Kv7 channel inhibitor XE-991 thereby providing support the functional specificity of XE-991 for Kv7 channels in our recording conditions.

### Inhibition of Kv7 channels enhances the ADP

Our data show that Kv7 channels do not affect NMDAR activity within EPSPs. However, active Kv7 channels may regulate NMDAR activity during LTP induction by reducing postsynaptic depolarisation in response to action potentials. The inhibition of Kv7 channels could either increase action potential duration and/or ADP amplitude [26], [28].

**Figure 4:**
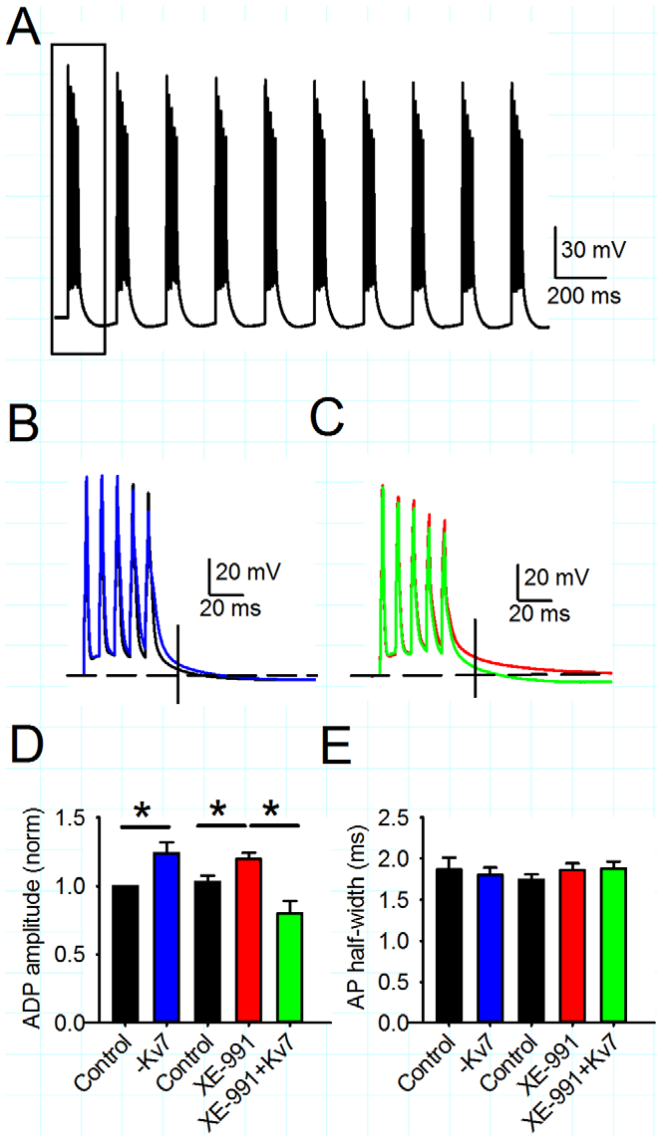
Inhibition or negation of Kv7 conductance increases the after-depolarisation. A) Example voltage trace for theta burst protocol of postsynaptic action potentials. Box illustrates section shown in B and C. B) Negative dynamic clamp Kv7 conductance (blue) increases the after-depolarisation (ADP) measured 20 ms after the last action potential (illustrated by vertical line) in a theta burst compared to control (black). C) XE-991 (10 µM, red) increases the ADP compared to control (black) and this was reversed by introduction of positive dynamic clamp Kv7 conductance (green). D) Average ADP amplitude was increased by negative dynamic clamp Kv7 conductance or XE-991. E) Average action potential (AP) half-width for the first action potential in a theta burst was unchanged by negative dynamic clamp Kv7 conductance or XE-991.

Application of XE-991 (10 µM) increased the ADP amplitude measured 20 ms after the last action potential of a burst of 5 action potentials (Figure 4C & D; ADP amplitude 1.20±0.05 fold versus control, n = 5, p<0.05). Similarly, removal of Kv7 conductance by dynamic clamp also increased the ADP amplitude (Figure 4B & D; ADP amplitude 1.24±0.08 fold versus control, n = 5, p<0.05). Importantly, the effect of XE-991 was fully reversed by subsequent introduction of Kv7 conductance (Figure 4C & D; ADP amplitude 0.80±0.09, n = 5, versus control p<0.05) indicating that Kv7 channels contribute to the membrane potential following a burst of action potentials, and their inhibition increases the ADP.

Kv7 channels have slow activation kinetics and thus are not usually considered to contribute to the membrane potential during action potentials [26]. However, they may contribute to action potential kinetics by changing input resistance. Analysis of action potential half-width following application of XE-991 revealed little change in the first action potential in the burst either with removal of Kv7 conductance or application of XE-991 (Figure 4E). Therefore, the major effect of Kv7 channel inhibition is on the post-spiking ADP amplitude rather than the action potentials themselves.

### Negation of Kv7 channels by dynamic clamp facilitates LTP induction

**Figure 5:**
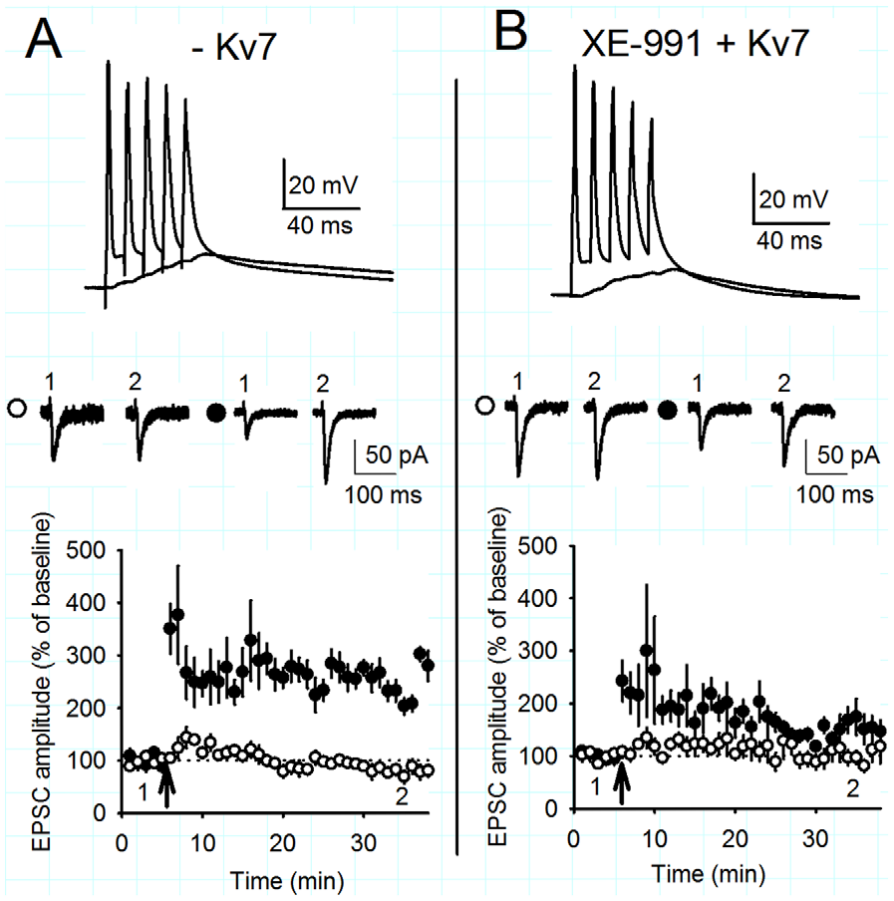
Negation of Kv7 by dynamic clamp facilitates the induction of LTP. A) Negative dynamic clamp Kv7 conductance facilitates LTP. Coincident TBP of subthreshold EPSPs and somatic action potentials induced pathway specific LTP in the test (black circles), but not in control (white circles) pathways. The arrow indicates the timing of the TBP protocol. Example voltage traces show the initial burst of 5 coincident EPSPs and action potentials and a single test burst of 5 subthreshold EPSPs. Example current traces from a single experiment illustrating the mean EPSC response during the baseline (1) and at 30–35 minutes (2) in the test and control pathways. B) Positive dynamic clamp conductance significantly diminished the amount of LTP in the presence of XE-991 (10 µM).

Our data shows that inhibition of Kv7 channels by XE-991 facilitates LTP induction and data using XE-991 and dynamic clamp suggests that the mechanism of facilitation is by increasing postsynaptic depolarisation during and after a burst of action potentials. Therefore, the use of dynamic clamp to mimic the inhibition of Kv7 current would also be expected to facilitate LTP, whereas reintroduction of a Kv7-like conductance after XE-991 would be expected to inhibit LTP induction. We confirmed the role of Kv7 channels in the facilitation of LTP by negating Kv7 conductance using dynamic clamp which produced significant pathway-specific LTP (Figure 5A; 240±14% vs. 77±7%, test vs. control pathway, n = 6, p<0.01). Furthermore, LTP was significantly reduced in the presence of XE-991 after replacement of Kv7 conductance with positive dynamic clamp (Figure 5B; 156±17% vs. 101±12%, test vs. control pathway, n = 6, p<0.05). These results confirm that inhibition of Kv7 channels facilitates the induction of LTP.

## Discussion

Activation of muscarinic receptors in the hippocampus produces two robust and consistent effects, namely modulation of ionic conductances, in particular inhibition of K^+^ channels, and the facilitation of LTP. Our experiments here have shown how muscarinic inhibition of Kv7 channels enhances intrinsic excitability of CA1 pyramidal cells and thus facilitates the induction of LTP, thus providing new evidence for a link between the two major facets of muscarinic activity in the hippocampus. We have demonstrated this link using dual pharmacological and physiological approaches to manipulate the Kv7 channel-mediated conductance in hippocampal CA1 pyramidal cells.

To facilitate LTP, we assume inhibition of Kv7 channels enhances the opening of NMDARs and therefore calcium influx into postsynaptic spines during TBP. There are two possible mechanisms by which this might occur. Similar to the situation for inhibition of KCa2 channels, the inhibition of Kv7 channels may act locally within dendritic spines to enhance NMDAR opening during synaptic transmission [6], [24]. Alternatively, inhibition of Kv7 channels may act globally to enhance the depolarisation that occurs during and after a burst of postsynaptic action potentials. The enhanced depolarisation would then increase NMDAR opening during coincident pre- and post-synaptic activity. Here we demonstrate that the NMDAR-mediated component of EPSPs following presynaptic stimulation is not increased by inhibition of Kv7 channels, arguing against a local mechanism of action within dendritic spines. Instead we show that inhibition of Kv7 enhances the ADP following a burst of postsynaptic action potentials [26] demonstrating that global cellular depolarisation is increased during TBP, which in turn will lead to increased NMDAR opening during coincident pre- and post-synaptic activity. This agrees with data showing that Kv7 channels on CA1 pyramidal cells are predominantly located perisomatically and not on distal dendrites [39], [40] and are thus ideally suited to the regulation of global excitability. Conversely, KCa2 channels have been shown to be located within spines and closely regulate NMDAR opening during synaptic transmission [24]. M_1_ muscarinic receptors are present on dendrites and dendritic spines [49], [50] and since they couple to Kv7 channels thought to be located predominantly on the axo-somatic membrane [40] it is assumed they are also found perisomatically although detailed immunohistochemical data is currently lacking. The inhibition of KCa2 channels located in spines together with Kv7 channels located perisomatically represent dual distinct mechanisms for the facilitation of LTP by activation of muscarinic receptors.

Inhibition of Kv7 channels by XE-991 or negation of Kv7 conductance with the use of dynamic clamp produced similar effects on cellular excitability and LTP induction. Furthermore, the effects of XE-991 could be reversed by reinstating Kv7 conductance with the dynamic clamp system. The use of these two diverse approaches that provide similar results supports the conclusion that Kv7 conductance is important for the facilitation of LTP by receptors which modulate Kv7 function, for example muscarinic receptors and other G-protein coupled receptors that signal via Gq and phospholipase C. Since Kv7 channels are preferentially located in the perisomatic region [39], [40], the conductance is particularly amenable to dynamic clamp applied through a somatic recording pipette. The use of dynamic clamp confirms that it is only postsynaptic inhibition of Kv7 channels that is required to enable LTP in our experiments.

Notably, as well as playing an important role in determining the ease with which LTP can be produced, Kv7-mediated conductances in hippocampal pyramidal cells exhibit considerable activity-dependent plasticity in their own right. For example, various patterns of intrinsic activity can seemingly increase the functional activity of Kv7 channels in hippocampal pyramidal cells [28], [51]. This is observed experimentally as a persistent XE-991-sensitive decrease in the ADP. Our data suggest such activity-dependent changes to Kv7-mediated conductances could also result in significant changes to the ease with which LTP can be induced in pyramidal cells.

The kinetics and voltage dependence for Kv7 channels that we have used for dynamic clamp experiments are derived from experimental data for heteromeric Kv7.2/7.3 channels [36], [37]. CA1 pyramidal cells express Kv7.2, Kv7.3 and Kv7.5 which have similar kinetics and voltage dependencies when expressed either as homomers or heteromers [52]. Thus we have replicated as closely as possible the conductance properties of Kv7 channels with the dynamic clamp system and demonstrate a successful reversal of pharmacological blockade.

Kv7 channels are key regulators of neuronal excitability and it has been shown that mutations in Kv7 channels are associated with some forms of epilepsy [53], [54] and Kv7 channel function also plays a role in persistent intrinsic plasticity [28], [51] and learning and memory [30], [31], [55], [56]. Our data support a role for neurotransmitter modulation of Kv7 channels in the induction of synaptic plasticity in the hippocampus, producing an additional route to the facilitation of LTP by muscarinic receptors over and above that generated by the inhibition of KCa2 channels in post-synaptic spines [6].
